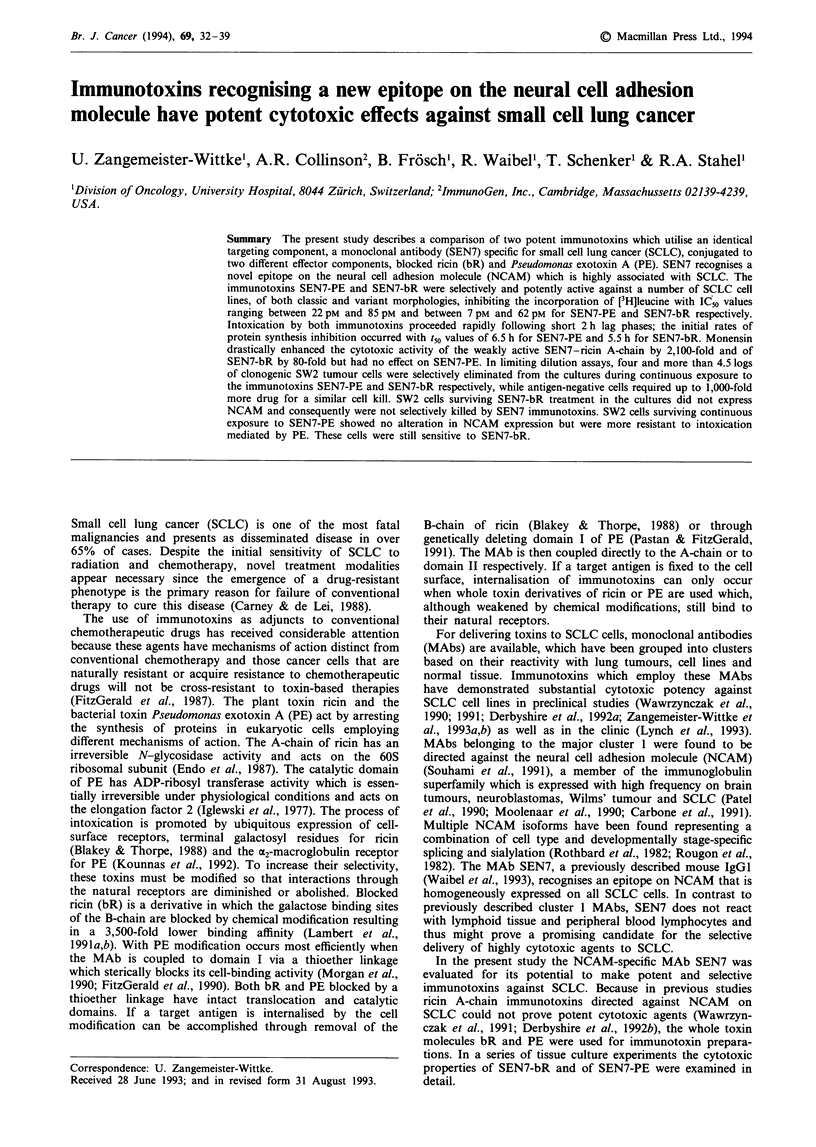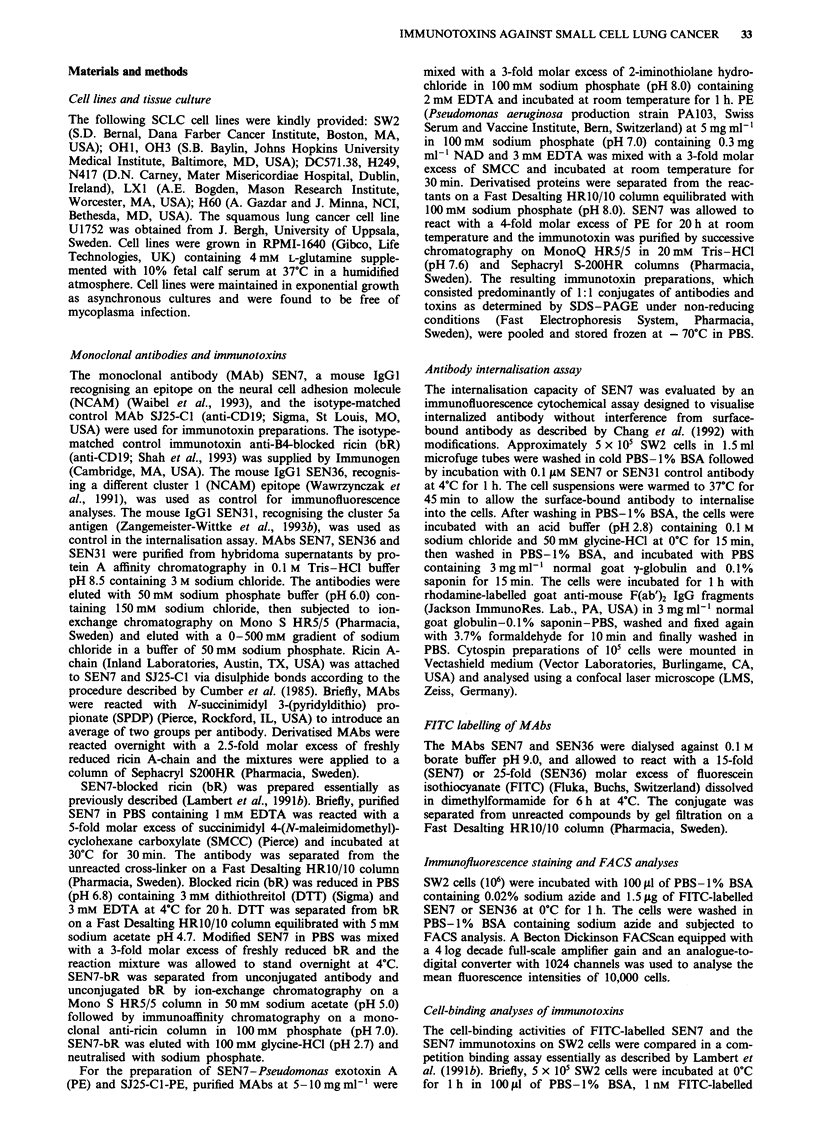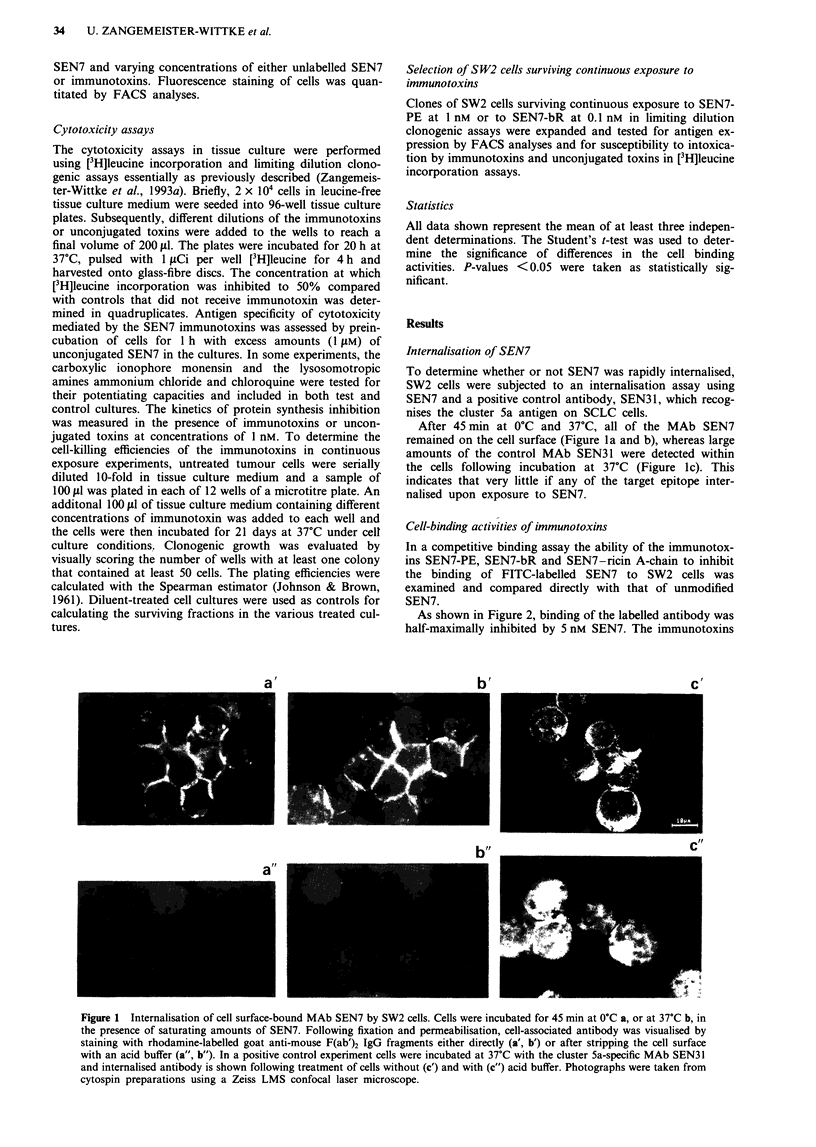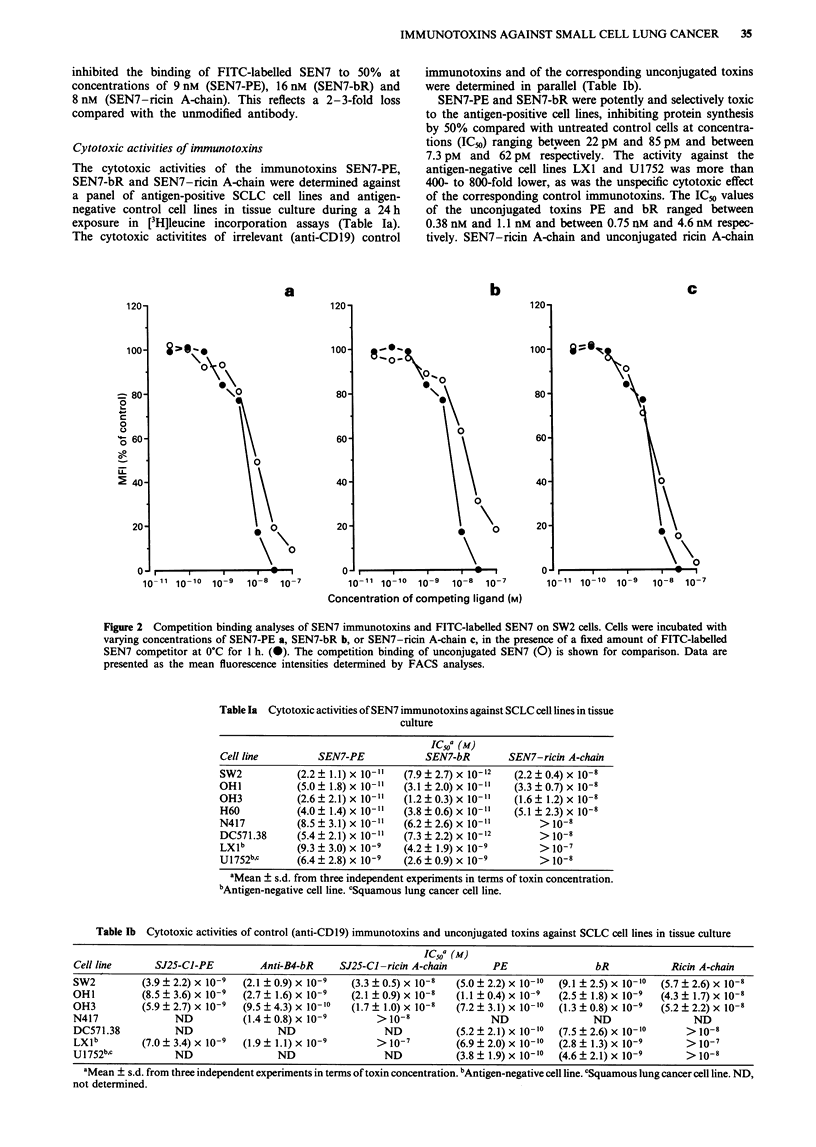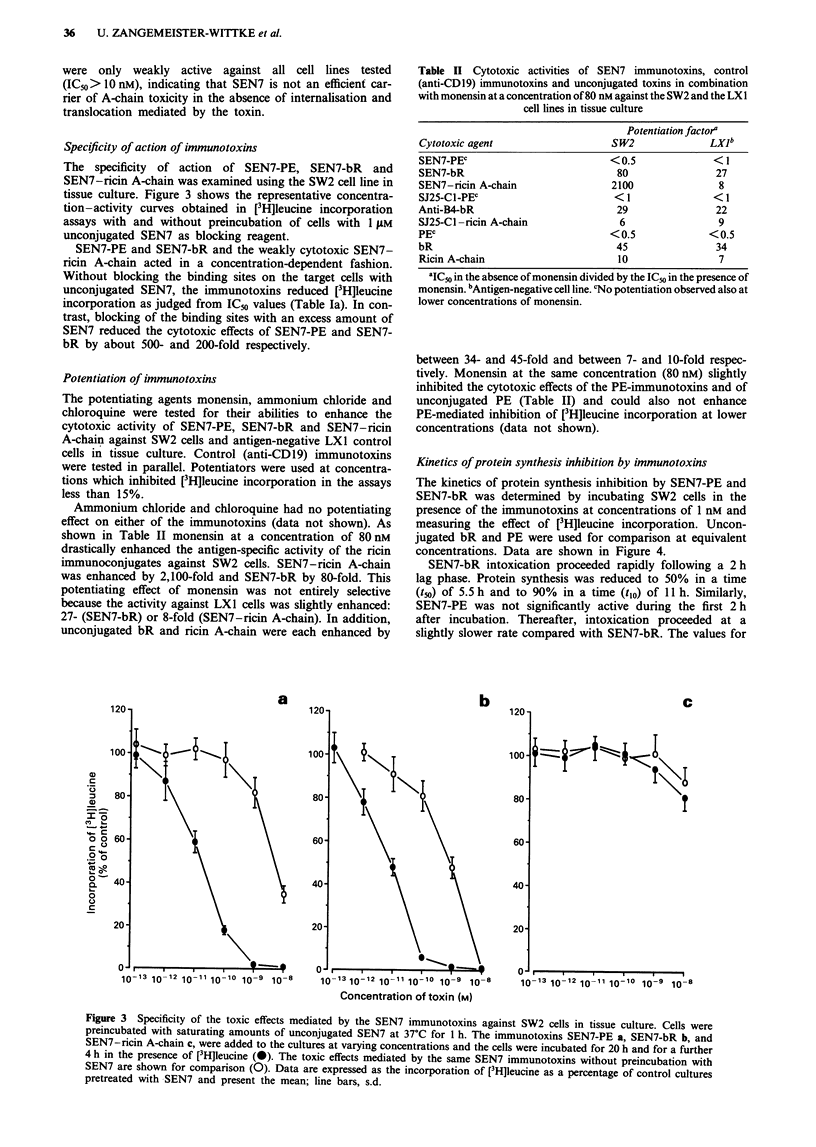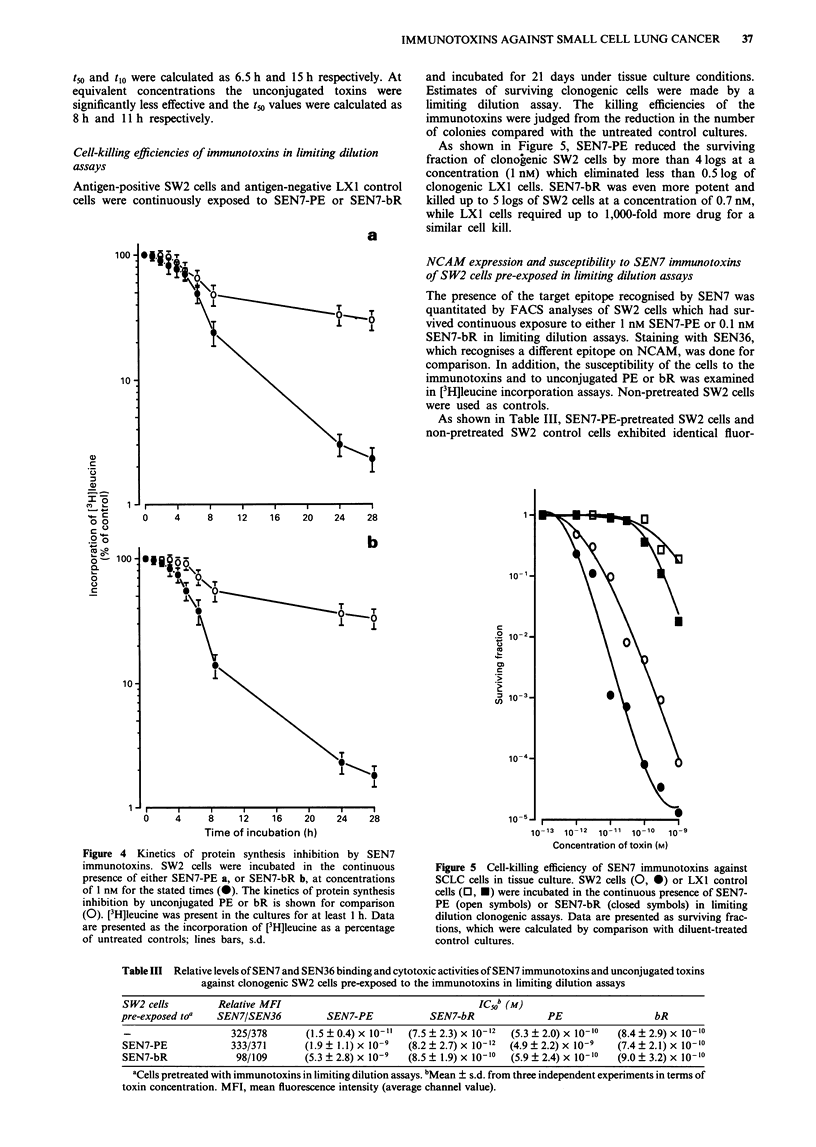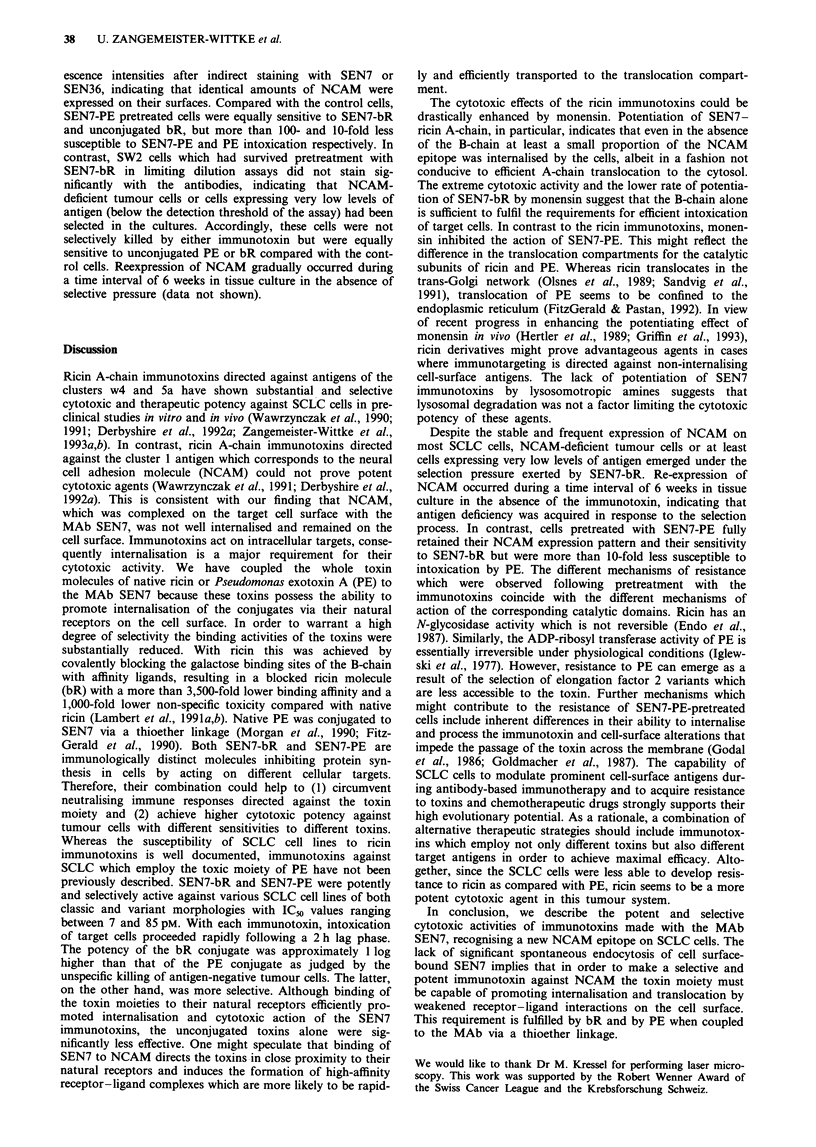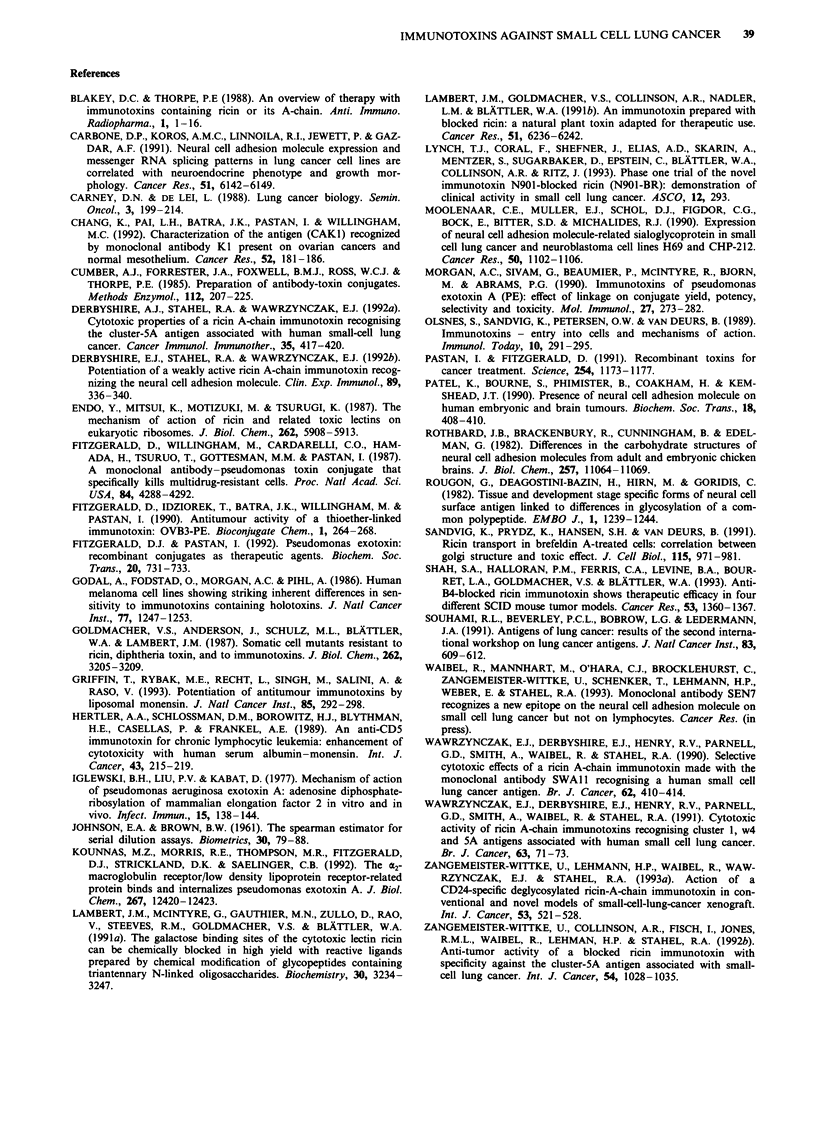# Immunotoxins recognising a new epitope on the neural cell adhesion molecule have potent cytotoxic effects against small cell lung cancer.

**DOI:** 10.1038/bjc.1994.5

**Published:** 1994-01

**Authors:** U. Zangemeister-Wittke, A. R. Collinson, B. Frösch, R. Waibel, T. Schenker, R. A. Stahel

**Affiliations:** Division of Oncology, University Hospital, Zürich, Switzerland.

## Abstract

**Images:**


Br. J. Cancer (1994), 69, 32-39 © Macmillan Press Ltd., 1994

### Immunotoxins recognising a new epitope on the neural cell adhesion molecule have potent cytotoxic effects against small cell lung cancer

U. Zangemeister-Wittke[1], A.R. Collinson[2], B. Frösch[1], R. Waibel[1], T. Schenker[1] & R.A. Stahel[1]

[1]Division of Oncology, University Hospital, 8044 Zürich, Switzerland; [2]ImmunoGen, Inc., Cambridge, Massachussetts 02139-4239, USA.

**Summary** The present study describes a comparison of two potent immunotoxins which utilise an identical targeting component, a monoclonal antibody (SEN7) specific for small cell lung cancer (SCLC), conjugated to two different effector components, blocked ricin (bR) and *Pseudomonas* exotoxin A (PE). SEN7 recognises a novel epitope on the neural cell adhesion molecule (NCAM) which is highly associated with SCLC. The immunotoxins SEN7-PE and SEN7-bR were selectively and potently active against a number of SCLC cell lines, of both classic and variant morphologies, inhibiting the incorporation of [$^3$H]leucine with $IC_{50}$ values ranging between 22 pM and 85 pM and between 7 pM and 62 pM for SEN7-PE and SEN7-bR respectively. Intoxication by both immunotoxins proceeded rapidly following short 2 h lag phases; the initial rates of protein synthesis inhibition occurred with $t_{50}$ values of 6.5 h for SEN7-PE and 5.5 h for SEN7-bR. Monensin drastically enhanced the cytotoxic activity of the weakly active SEN7-ricin A-chain by 2,100-fold and of SEN7-bR by 80-fold but had no effect on SEN7-PE. In limiting dilution assays, four and more than 4.5 logs of clonogenic SW2 tumour cells were selectively eliminated from the cultures during continuous exposure to the immunotoxins SEN7-PE and SEN7-bR respectively, while antigen-negative cells required up to 1,000-fold more drug for a similar cell kill. SW2 cells surviving SEN7-bR treatment in the cultures did not express NCAM and consequently were not selectively killed by SEN7 immunotoxins. SW2 cells surviving continuous exposure to SEN7-PE showed no alteration in NCAM expression but were more resistant to intoxication mediated by PE. These cells were still sensitive to SEN7-bR.

Small cell lung cancer (SCLC) is one of the most fatal malignancies and presents as disseminated disease in over 65% of cases. Despite the initial sensitivity of SCLC to radiation and chemotherapy, novel treatment modalities appear necessary since the emergence of a drug-resistant phenotype is the primary reason for failure of conventional therapy to cure this disease (Carney & de Lei, 1988).

The use of immunotoxins as adjuncts to conventional chemotherapeutic drugs has received considerable attention because these agents have mechanisms of action distinct from conventional chemotherapy and those cancer cells that are naturally resistant or acquire resistance to chemotherapeutic drugs will not be cross-resistant to toxin-based therapies (FitzGerald *et al.*, 1987). The plant toxin ricin and the bacterial toxin *Pseudomonas* exotoxin A (PE) act by arresting the synthesis of proteins in eukaryotic cells employing different mechanisms of action. The A-chain of ricin has an irreversible *N*-glycosidase activity and acts on the 60S ribosomal subunit (Endo *et al.*, 1987). The catalytic domain of PE has ADP-ribosyl transferase activity which is essentially irreversible under physiological conditions and acts on the elongation factor 2 (Iglewski *et al.*, 1977). The process of intoxication is promoted by ubiquitous expression of cell-surface receptors, terminal galactosyl residues for ricin (Blakey & Thorpe, 1988) and the $\alpha_2$-macroglobulin receptor for PE (Kounnas *et al.*, 1992). To increase their selectivity, these toxins must be modified so that interactions through the natural receptors are diminished or abolished. Blocked ricin (bR) is a derivative in which the galactose binding sites of the B-chain are blocked by chemical modification resulting in a 3,500-fold lower binding affinity (Lambert *et al.*, 1991*a*,*b*). With PE modification occurs most efficiently when the MAb is coupled to domain I via a thioether linkage which sterically blocks its cell-binding activity (Morgan *et al.*, 1990; FitzGerald *et al.*, 1990). Both bR and PE blocked by a thioether linkage have intact translocation and catalytic domains. If a target antigen is internalised by the cell modification can be accomplished through removal of the B-chain of ricin (Blakey & Thorpe, 1988) or through genetically deleting domain I of PE (Pastan & FitzGerald, 1991). The MAb is then coupled directly to the A-chain or to domain II respectively. If a target antigen is fixed to the cell surface, internalisation of immunotoxins can only occur when whole toxin derivatives of ricin or PE are used which, although weakened by chemical modifications, still bind to their natural receptors.

For delivering toxins to SCLC cells, monoclonal antibodies (MAbs) are available, which have been grouped into clusters based on their reactivity with lung tumours, cell lines and normal tissue. Immunotoxins which employ these MAbs have demonstrated substantial cytotoxic potency against SCLC cell lines in preclinical studies (Wawrzynczak *et al.*, 1990; 1991; Derbyshire *et al.*, 1992*a*; Zangemeister-Wittke *et al.*, 1993*a*,*b*) as well as in the clinic (Lynch *et al.*, 1993). MAbs belonging to the major cluster 1 were found to be directed against the neural cell adhesion molecule (NCAM) (Souhami *et al.*, 1991), a member of the immunoglobulin superfamily which is expressed with high frequency on brain tumours, neuroblastomas, Wilms' tumour and SCLC (Patel *et al.*, 1990; Moolenaar *et al.*, 1990; Carbone *et al.*, 1991). Multiple NCAM isoforms have been found representing a combination of cell type and developmentally stage-specific splicing and sialylation (Rothbard *et al.*, 1982; Rougon *et al.*, 1982). The MAb SEN7, a previously described mouse IgG1 (Waibel *et al.*, 1993), recognises an epitope on NCAM that is homogeneously expressed on all SCLC cells. In contrast to previously described cluster 1 MAbs, SEN7 does not react with lymphoid tissue and peripheral blood lymphocytes and thus might prove a promising candidate for the selective delivery of highly cytotoxic agents to SCLC.

In the present study the NCAM-specific MAb SEN7 was evaluated for its potential to make potent and selective immunotoxins against SCLC. Because in previous studies ricin A-chain immunotoxins directed against NCAM on SCLC could not prove potent cytotoxic agents (Wawrzynczak *et al.*, 1991; Derbyshire *et al.*, 1992*b*), the whole toxin molecules bR and PE were used for immunotoxin preparations. In a series of tissue culture experiments the cytotoxic properties of SEN7-bR and of SEN7-PE were examined in detail.

Correspondence: U. Zangemeister-Wittke.
Received 28 June 1993; and in revised form 31 August 1993.

### Materials and methods

### *Cell lines and tissue culture*

The following SCLC cell lines were kindly provided: SW2 (S.D. Bernal, Dana Farber Cancer Institute, Boston, MA, USA); OH1, OH3 (S.B. Baylin, Johns Hopkins University Medical Institute, Baltimore, MD, USA); DC571.38, H249, N417 (D.N. Carney, Mater Misericordiae Hospital, Dublin, Ireland), LX1 (A.E. Bogden, Mason Research Institute, Worcester, MA, USA); H60 (A. Gazdar and J. Minna, NCI, Bethesda, MD, USA). The squamous lung cancer cell line U1752 was obtained from J. Bergh, University of Uppsala, Sweden. Cell lines were grown in RPMI-1640 (Gibco, Life Technologies, UK) containing 4 mM L-glutamine supplemented with 10% fetal calf serum at 37°C in a humidified atmosphere. Cell lines were maintained in exponential growth as asynchronous cultures and were found to be free of mycoplasma infection.

### *Monoclonal antibodies and immunotoxins*

The monoclonal antibody (MAb) SEN7, a mouse IgG1 recognising an epitope on the neural cell adhesion molecule (NCAM) (Waibel *et al.*, 1993), and the isotype-matched control MAb SJ25-C1 (anti-CD19; Sigma, St Louis, MO, USA) were used for immunotoxin preparations. The isotype-matched control immunotoxin anti-B4-blocked ricin (bR) (anti-CD19; Shah *et al.*, 1993) was supplied by Immunogen (Cambridge, MA, USA). The mouse IgG1 SEN36, recognising a different cluster 1 (NCAM) epitope (Wawrzynczak *et al.*, 1991), was used as control for immunofluorescence analyses. The mouse IgG1 SEN31, recognising the cluster 5a antigen (Zangemeister-Wittke *et al.*, 1993*b*), was used as control in the internalisation assay. MAbs SEN7, SEN36 and SEN31 were purified from hybridoma supernatants by protein A affinity chromatography in 0.1 M Tris–HCl buffer pH 8.5 containing 3 M sodium chloride. The antibodies were eluted with 50 mM sodium phosphate buffer (pH 6.0) containing 150 mM sodium chloride, then subjected to ion-exchange chromatography on Mono S HR5/5 (Pharmacia, Sweden) and eluted with a 0–500 mM gradient of sodium chloride in a buffer of 50 mM sodium phosphate. Ricin A-chain (Inland Laboratories, Austin, TX, USA) was attached to SEN7 and SJ25-C1 via disulphide bonds according to the procedure described by Cumber *et al.* (1985). Briefly, MAbs were reacted with *N*-succinimidyl 3-(pyridyldithio) propionate (SPDP) (Pierce, Rockford, IL, USA) to introduce an average of two groups per antibody. Derivatised MAbs were reacted overnight with a 2.5-fold molar excess of freshly reduced ricin A-chain and the mixtures were applied to a column of Sephacryl S200HR (Pharmacia, Sweden).

SEN7-blocked ricin (bR) was prepared essentially as previously described (Lambert *et al.*, 1991*b*). Briefly, purified SEN7 in PBS containing 1 mM EDTA was reacted with a 5-fold molar excess of succinimidyl 4-(*N*-maleimidomethyl)-cyclohexane carboxylate (SMCC) (Pierce) and incubated at 30°C for 30 min. The antibody was separated from the unreacted cross-linker on a Fast Desalting HR10/10 column (Pharmacia, Sweden). Blocked ricin (bR) was reduced in PBS (pH 6.8) containing 3 mM dithiothreitol (DTT) (Sigma) and 3 mM EDTA at 4°C for 20 h. DTT was separated from bR on a Fast Desalting HR10/10 column equilibrated with 5 mM sodium acetate pH 4.7. Modified SEN7 in PBS was mixed with a 3-fold molar excess of freshly reduced bR and the reaction mixture was allowed to stand overnight at 4°C. SEN7-bR was separated from unconjugated antibody and unconjugated bR by ion-exchange chromatography on a Mono S HR5/5 column in 50 mM sodium acetate (pH 5.0) followed by immunoaffinity chromatography on a monoclonal anti-ricin column in 100 mM phosphate (pH 7.0). SEN7-bR was eluted with 100 mM glycine-HCl (pH 2.7) and neutralised with sodium phosphate.

For the preparation of SEN7–*Pseudomonas* exotoxin A (PE) and SJ25-C1-PE, purified MAbs at 5–10 mg ml$^{-1}$ were mixed with a 3-fold molar excess of 2-iminothiolane hydrochloride in 100 mM sodium phosphate (pH 8.0) containing 2 mM EDTA and incubated at room temperature for 1 h. PE (*Pseudomonas aeruginosa* production strain PA103, Swiss Serum and Vaccine Institute, Bern, Switzerland) at 5 mg ml$^{-1}$ in 100 mM sodium phosphate (pH 7.0) containing 0.3 mg ml$^{-1}$ NAD and 3 mM EDTA was mixed with a 3-fold molar excess of SMCC and incubated at room temperature for 30 min. Derivatised proteins were separated from the reactants on a Fast Desalting HR10/10 column equilibrated with 100 mM sodium phosphate (pH 8.0). SEN7 was allowed to react with a 4-fold molar excess of PE for 20 h at room temperature and the immunotoxin was purified by successive chromatography on MonoQ HR5/5 in 20 mM Tris–HCl (pH 7.6) and Sephacryl S-200HR columns (Pharmacia, Sweden). The resulting immunotoxin preparations, which consisted predominantly of 1:1 conjugates of antibodies and toxins as determined by SDS–PAGE under non-reducing conditions (Fast Electrophoresis System, Pharmacia, Sweden), were pooled and stored frozen at – 70°C in PBS.

### *Antibody internalisation assay*

The internalisation capacity of SEN7 was evaluated by an immunofluorescence cytochemical assay designed to visualise internalized antibody without interference from surface-bound antibody as described by Chang *et al.* (1992) with modifications. Approximately $5 \times 10^5$ SW2 cells in 1.5 ml microfuge tubes were washed in cold PBS–1% BSA followed by incubation with 0.1 μM SEN7 or SEN31 control antibody at 4°C for 1 h. The cell suspensions were warmed to 37°C for 45 min to allow the surface-bound antibody to internalise into the cells. After washing in PBS–1% BSA, the cells were incubated with an acid buffer (pH 2.8) containing 0.1 M sodium chloride and 50 mM glycine-HCl at 0°C for 15 min, then washed in PBS–1% BSA, and incubated with PBS containing 3 mg ml$^{-1}$ normal goat γ-globulin and 0.1% saponin for 15 min. The cells were incubated for 1 h with rhodamine-labelled goat anti-mouse F(ab')$_2$ IgG fragments (Jackson ImmunoRes. Lab., PA, USA) in 3 mg ml$^{-1}$ normal goat globulin–0.1% saponin–PBS, washed and fixed again with 3.7% formaldehyde for 10 min and finally washed in PBS. Cytospin preparations of $10^5$ cells were mounted in Vectashield medium (Vector Laboratories, Burlingame, CA, USA) and analysed using a confocal laser microscope (LMS, Zeiss, Germany).

### *FITC labelling of MAbs*

The MAbs SEN7 and SEN36 were dialysed against 0.1 M borate buffer pH 9.0, and allowed to react with a 15-fold (SEN7) or 25-fold (SEN36) molar excess of fluorescein isothiocyanate (FITC) (Fluka, Buchs, Switzerland) dissolved in dimethylformamide for 6 h at 4°C. The conjugate was separated from unreacted compounds by gel filtration on a Fast Desalting HR10/10 column (Pharmacia, Sweden).

### *Immunofluorescence staining and FACS analyses*

SW2 cells ($10^6$) were incubated with 100 μl of PBS–1% BSA containing 0.02% sodium azide and 1.5 μg of FITC-labelled SEN7 or SEN36 at 0°C for 1 h. The cells were washed in PBS–1% BSA containing sodium azide and subjected to FACS analysis. A Becton Dickinson FACScan equipped with a 4 log decade full-scale amplifier gain and an analogue-to-digital converter with 1024 channels was used to analyse the mean fluorescence intensities of 10,000 cells.

### *Cell-binding analyses of immunotoxins*

The cell-binding activities of FITC-labelled SEN7 and the SEN7 immunotoxins on SW2 cells were compared in a competition binding assay essentially as described by Lambert *et al.* (1991*b*). Briefly, $5 \times 10^5$ SW2 cells were incubated at 0°C for 1 h in 100 μl of PBS–1% BSA, 1 nM FITC-labelled

SEN7 and varying concentrations of either unlabelled SEN7 or immunotoxins. Fluorescence staining of cells was quantitated by FACS analyses.

*Cytotoxicity assays*

The cytotoxicity assays in tissue culture were performed using [$^3$H]leucine incorporation and limiting dilution clonogenic assays essentially as previously described (Zangemeister-Wittke *et al.*, 1993*a*). Briefly, $2 \times 10^4$ cells in leucine-free tissue culture medium were seeded into 96-well tissue culture plates. Subsequently, different dilutions of the immunotoxins or unconjugated toxins were added to the wells to reach a final volume of 200 µl. The plates were incubated for 20 h at 37°C, pulsed with 1 µCi per well [$^3$H]leucine for 4 h and harvested onto glass-fibre discs. The concentration at which [$^3$H]leucine incorporation was inhibited to 50% compared with controls that did not receive immunotoxin was determined in quadruplicates. Antigen specificity of cytotoxicity mediated by the SEN7 immunotoxins was assessed by preincubation of cells for 1 h with excess amounts (1 µM) of unconjugated SEN7 in the cultures. In some experiments, the carboxylic ionophore monensin and the lysosomotropic amines ammonium chloride and chloroquine were tested for their potentiating capacities and included in both test and control cultures. The kinetics of protein synthesis inhibition was measured in the presence of immunotoxins or unconjugated toxins at concentrations of 1 nM. To determine the cell-killing efficiencies of the immunotoxins in continuous exposure experiments, untreated tumour cells were serially diluted 10-fold in tissue culture medium and a sample of 100 µl was plated in each of 12 wells of a microtitre plate. An additonal 100 µl of tissue culture medium containing different concentrations of immunotoxin was added to each well and the cells were then incubated for 21 days at 37°C under cell culture conditions. Clonogenic growth was evaluated by visually scoring the number of wells with at least one colony that contained at least 50 cells. The plating efficiencies were calculated with the Spearman estimator (Johnson & Brown, 1961). Diluent-treated cell cultures were used as controls for calculating the surviving fractions in the various treated cultures.

*Selection of SW2 cells surviving continuous exposure to immunotoxins*

Clones of SW2 cells surviving continuous exposure to SEN7-PE at 1 nM or to SEN7-bR at 0.1 nM in limiting dilution clonogenic assays were expanded and tested for antigen expression by FACS analyses and for susceptibility to intoxication by immunotoxins and unconjugated toxins in [$^3$H]leucine incorporation assays.

*Statistics*

All data shown represent the mean of at least three independent determinations. The Student's *t*-test was used to determine the significance of differences in the cell binding activities. *P*-values $<0.05$ were taken as statistically significant.

### Results

*Internalisation of SEN7*

To determine whether or not SEN7 was rapidly internalised, SW2 cells were subjected to an internalisation assay using SEN7 and a positive control antibody, SEN31, which recognises the cluster 5a antigen on SCLC cells.

After 45 min at 0°C and 37°C, all of the MAb SEN7 remained on the cell surface (Figure 1a and b), whereas large amounts of the control MAb SEN31 were detected within the cells following incubation at 37°C (Figure 1c). This indicates that very little if any of the target epitope internalised upon exposure to SEN7.

*Cell-binding activities of immunotoxins*

In a competitive binding assay the ability of the immunotoxins SEN7-PE, SEN7-bR and SEN7-ricin A-chain to inhibit the binding of FITC-labelled SEN7 to SW2 cells was examined and compared directly with that of unmodified SEN7.

As shown in Figure 2, binding of the labelled antibody was half-maximally inhibited by 5 nM SEN7. The immunotoxins

**Figure 1** Internalisation of cell surface-bound MAb SEN7 by SW2 cells. Cells were incubated for 45 min at 0°C **a**, or at 37°C **b**, in the presence of saturating amounts of SEN7. Following fixation and permeabilisation, cell-associated antibody was visualised by staining with rhodamine-labelled goat anti-mouse F(ab′)$_2$ IgG fragments either directly (**a′**, **b′**) or after stripping the cell surface with an acid buffer (**a″**, **b″**). In a positive control experiment cells were incubated at 37°C with the cluster 5a-specific MAb SEN31 and internalised antibody is shown following treatment of cells without (**c′**) and with (**c″**) acid buffer. Photographs were taken from cytospin preparations using a Zeiss LMS confocal laser microscope.

inhibited the binding of FITC-labelled SEN7 to 50% at concentrations of 9 nM (SEN7-PE), 16 nM (SEN7-bR) and 8 nM (SEN7–ricin A-chain). This reflects a 2–3-fold loss compared with the unmodified antibody.

### *Cytotoxic activities of immunotoxins*

The cytotoxic activities of the immunotoxins SEN7-PE, SEN7-bR and SEN7–ricin A-chain were determined against a panel of antigen-positive SCLC cell lines and antigen-negative control cell lines in tissue culture during a 24 h exposure in [$^3$H]leucine incorporation assays (Table Ia). The cytotoxic activitites of irrelevant (anti-CD19) control immunotoxins and of the corresponding unconjugated toxins were determined in parallel (Table Ib).

SEN7-PE and SEN7-bR were potently and selectively toxic to the antigen-positive cell lines, inhibiting protein synthesis by 50% compared with untreated control cells at concentrations ($IC_{50}$) ranging between 22 pM and 85 pM and between 7.3 pM and 62 pM respectively. The activity against the antigen-negative cell lines LX1 and U1752 was more than 400- to 800-fold lower, as was the unspecific cytotoxic effect of the corresponding control immunotoxins. The $IC_{50}$ values of the unconjugated toxins PE and bR ranged between 0.38 nM and 1.1 nM and between 0.75 nM and 4.6 nM respectively. SEN7–ricin A-chain and unconjugated ricin A-chain

**Figure 2** Competition binding analyses of SEN7 immunotoxins and FITC-labelled SEN7 on SW2 cells. Cells were incubated with varying concentrations of SEN7-PE **a**, SEN7-bR **b**, or SEN7–ricin A-chain **c**, in the presence of a fixed amount of FITC-labelled SEN7 competitor at 0°C for 1 h. (●). The competition binding of unconjugated SEN7 (○) is shown for comparison. Data are presented as the mean fluorescence intensities determined by FACS analyses.

**Table Ia** Cytotoxic activities of SEN7 immunotoxins against SCLC cell lines in tissue culture

| Cell line | $IC_{50}{}^a$ (M) SEN7-PE | SEN7-bR | SEN7–ricin A-chain |
|---|---|---|---|
| SW2 | $(2.2 \pm 1.1) \times 10^{-11}$ | $(7.9 \pm 2.7) \times 10^{-12}$ | $(2.2 \pm 0.4) \times 10^{-8}$ |
| OH1 | $(5.0 \pm 1.8) \times 10^{-11}$ | $(3.1 \pm 2.0) \times 10^{-11}$ | $(3.3 \pm 0.7) \times 10^{-8}$ |
| OH3 | $(2.6 \pm 2.1) \times 10^{-11}$ | $(1.2 \pm 0.3) \times 10^{-11}$ | $(1.6 \pm 1.2) \times 10^{-8}$ |
| H60 | $(4.0 \pm 1.4) \times 10^{-11}$ | $(3.8 \pm 0.6) \times 10^{-11}$ | $(5.1 \pm 2.3) \times 10^{-8}$ |
| N417 | $(8.5 \pm 3.1) \times 10^{-11}$ | $(6.2 \pm 2.6) \times 10^{-11}$ | $>10^{-8}$ |
| DC571.38 | $(5.4 \pm 2.1) \times 10^{-11}$ | $(7.3 \pm 2.2) \times 10^{-12}$ | $>10^{-8}$ |
| LX1[b] | $(9.3 \pm 3.0) \times 10^{-9}$ | $(4.2 \pm 1.9) \times 10^{-9}$ | $>10^{-7}$ |
| U1752[b,c] | $(6.4 \pm 2.8) \times 10^{-9}$ | $(2.6 \pm 0.9) \times 10^{-9}$ | $>10^{-8}$ |

[a]Mean ± s.d. from three independent experiments in terms of toxin concentration. [b]Antigen-negative cell line. [c]Squamous lung cancer cell line.

**Table Ib** Cytotoxic activities of control (anti-CD19) immunotoxins and unconjugated toxins against SCLC cell lines in tissue culture

| Cell line | $IC_{50}{}^a$ (M) SJ25-C1-PE | Anti-B4-bR | SJ25-C1–ricin A-chain | PE | bR | Ricin A-chain |
|---|---|---|---|---|---|---|
| SW2 | $(3.9 \pm 2.2) \times 10^{-9}$ | $(2.1 \pm 0.9) \times 10^{-9}$ | $(3.3 \pm 0.5) \times 10^{-8}$ | $(5.0 \pm 2.2) \times 10^{-10}$ | $(9.1 \pm 2.5) \times 10^{-10}$ | $(5.7 \pm 2.6) \times 10^{-8}$ |
| OH1 | $(8.5 \pm 3.6) \times 10^{-9}$ | $(2.7 \pm 1.6) \times 10^{-9}$ | $(2.1 \pm 0.9) \times 10^{-8}$ | $(1.1 \pm 0.4) \times 10^{-9}$ | $(2.5 \pm 1.8) \times 10^{-9}$ | $(4.3 \pm 1.7) \times 10^{-8}$ |
| OH3 | $(5.9 \pm 2.7) \times 10^{-9}$ | $(9.5 \pm 4.3) \times 10^{-10}$ | $(1.7 \pm 1.0) \times 10^{-8}$ | $(7.2 \pm 3.1) \times 10^{-10}$ | $(1.3 \pm 0.8) \times 10^{-9}$ | $(5.2 \pm 2.2) \times 10^{-8}$ |
| N417 | ND | $(1.4 \pm 0.8) \times 10^{-9}$ | $>10^{-8}$ | ND | ND | ND |
| DC571.38 | ND | ND | ND | $(5.2 \pm 2.1) \times 10^{-10}$ | $(7.5 \pm 2.6) \times 10^{-10}$ | $>10^{-8}$ |
| LX1[b] | $(7.0 \pm 3.4) \times 10^{-9}$ | $(1.9 \pm 1.1) \times 10^{-9}$ | $>10^{-7}$ | $(6.9 \pm 2.0) \times 10^{-10}$ | $(2.8 \pm 1.3) \times 10^{-9}$ | $>10^{-7}$ |
| U1752[b,c] | ND | ND | ND | $(3.8 \pm 1.9) \times 10^{-10}$ | $(4.6 \pm 2.1) \times 10^{-9}$ | $>10^{-8}$ |

[a]Mean ± s.d. from three independent experiments in terms of toxin concentration. [b]Antigen-negative cell line. [c]Squamous lung cancer cell line. ND, not determined.

were only weakly active against all cell lines tested ($IC_{50}$ > 10 nM), indicating that SEN7 is not an efficient carrier of A-chain toxicity in the absence of internalisation and translocation mediated by the toxin.

*Specificity of action of immunotoxins*

The specificity of action of SEN7-PE, SEN7-bR and SEN7-ricin A-chain was examined using the SW2 cell line in tissue culture. Figure 3 shows the representative concentration-activity curves obtained in [$^3$H]leucine incorporation assays with and without preincubation of cells with 1 μM unconjugated SEN7 as blocking reagent.

SEN7-PE and SEN7-bR and the weakly cytotoxic SEN7-ricin A-chain acted in a concentration-dependent fashion. Without blocking the binding sites on the target cells with unconjugated SEN7, the immunotoxins reduced [$^3$H]leucine incorporation as judged from $IC_{50}$ values (Table Ia). In contrast, blocking of the binding sites with an excess amount of SEN7 reduced the cytotoxic effects of SEN7-PE and SEN7-bR by about 500- and 200-fold respectively.

*Potentiation of immunotoxins*

The potentiating agents monensin, ammonium chloride and chloroquine were tested for their abilities to enhance the cytotoxic activity of SEN7-PE, SEN7-bR and SEN7-ricin A-chain against SW2 cells and antigen-negative LX1 control cells in tissue culture. Control (anti-CD19) immunotoxins were tested in parallel. Potentiators were used at concentrations which inhibited [$^3$H]leucine incorporation in the assays less than 15%.

Ammonium chloride and chloroquine had no potentiating effect on either of the immunotoxins (data not shown). As shown in Table II monensin at a concentration of 80 nM drastically enhanced the antigen-specific activity of the ricin immunoconjugates against SW2 cells. SEN7-ricin A-chain was enhanced by 2,100-fold and SEN7-bR by 80-fold. This potentiating effect of monensin was not entirely selective because the activity against LX1 cells was slightly enhanced: 27- (SEN7-bR) or 8-fold (SEN7-ricin A-chain). In addition, unconjugated bR and ricin A-chain were each enhanced by between 34- and 45-fold and between 7- and 10-fold respectively. Monensin at the same concentration (80 nM) slightly inhibited the cytotoxic effects of the PE-immunotoxins and of unconjugated PE (Table II) and could also not enhance PE-mediated inhibition of [$^3$H]leucine incorporation at lower concentrations (data not shown).

**Table II** Cytotoxic activities of SEN7 immunotoxins, control (anti-CD19) immunotoxins and unconjugated toxins in combination with monensin at a concentration of 80 nM against the SW2 and the LX1 cell lines in tissue culture

| | *Potentiation factor*[a] | |
|---|---|---|
| *Cytotoxic agent* | *SW2* | *LX1*[b] |
| SEN7-PE[c] | <0.5 | <1 |
| SEN7-bR | 80 | 27 |
| SEN7-ricin A-chain | 2100 | 8 |
| SJ25-C1-PE[c] | <1 | <1 |
| Anti-B4-bR | 29 | 22 |
| SJ25-C1-ricin A-chain | 6 | 9 |
| PE[c] | <0.5 | <0.5 |
| bR | 45 | 34 |
| Ricin A-chain | 10 | 7 |

[a]$IC_{50}$ in the absence of monensin divided by the $IC_{50}$ in the presence of monensin. [b]Antigen-negative cell line. [c]No potentiation observed also at lower concentrations of monensin.

*Kinetics of protein synthesis inhibition by immunotoxins*

The kinetics of protein synthesis inhibition by SEN7-PE and SEN7-bR was determined by incubating SW2 cells in the presence of the immunotoxins at concentrations of 1 nM and measuring the effect of [$^3$H]leucine incorporation. Unconjugated bR and PE were used for comparison at equivalent concentrations. Data are shown in Figure 4.

SEN7-bR intoxication proceeded rapidly following a 2 h lag phase. Protein synthesis was reduced to 50% in a time ($t_{50}$) of 5.5 h and to 90% in a time ($t_{10}$) of 11 h. Similarly, SEN7-PE was not significantly active during the first 2 h after incubation. Thereafter, intoxication proceeded at a slightly slower rate compared with SEN7-bR. The values for

**Figure 3** Specificity of the toxic effects mediated by the SEN7 immunotoxins against SW2 cells in tissue culture. Cells were preincubated with saturating amounts of unconjugated SEN7 at 37°C for 1 h. The immunotoxins SEN7-PE **a**, SEN7-bR **b**, and SEN7-ricin A-chain **c**, were added to the cultures at varying concentrations and the cells were incubated for 20 h and for a further 4 h in the presence of [$^3$H]leucine (●). The toxic effects mediated by the same SEN7 immunotoxins without preincubation with SEN7 are shown for comparison (○). Data are expressed as the incorporation of [$^3$H]leucine as a percentage of control cultures pretreated with SEN7 and present the mean; line bars, s.d.

$t_{50}$ and $t_{10}$ were calculated as 6.5 h and 15 h respectively. At equivalent concentrations the unconjugated toxins were significantly less effective and the $t_{50}$ values were calculated as 8 h and 11 h respectively.

*Cell-killing efficiencies of immunotoxins in limiting dilution assays*

Antigen-positive SW2 cells and antigen-negative LX1 control cells were continuously exposed to SEN7-PE or SEN7-bR and incubated for 21 days under tissue culture conditions. Estimates of surviving clonogenic cells were made by a limiting dilution assay. The killing efficiencies of the immunotoxins were judged from the reduction in the number of colonies compared with the untreated control cultures.

As shown in Figure 5, SEN7-PE reduced the surviving fraction of clonogenic SW2 cells by more than 4 logs at a concentration (1 nM) which eliminated less than 0.5 log of clonogenic LX1 cells. SEN7-bR was even more potent and killed up to 5 logs of SW2 cells at a concentration of 0.7 nM, while LX1 cells required up to 1,000-fold more drug for a similar cell kill.

*NCAM expression and susceptibility to SEN7 immunotoxins of SW2 cells pre-exposed in limiting dilution assays*

The presence of the target epitope recognised by SEN7 was quantitated by FACS analyses of SW2 cells which had survived continuous exposure to either 1 nM SEN7-PE or 0.1 nM SEN7-bR in limiting dilution assays. Staining with SEN36, which recognises a different epitope on NCAM, was done for comparison. In addition, the susceptibility of the cells to the immunotoxins and to unconjugated PE or bR was examined in [$^3$H]leucine incorporation assays. Non-pretreated SW2 cells were used as controls.

As shown in Table III, SEN7-PE-pretreated SW2 cells and non-pretreated SW2 control cells exhibited identical fluor-

**Figure 4** Kinetics of protein synthesis inhibition by SEN7 immunotoxins. SW2 cells were incubated in the continuous presence of either SEN7-PE **a**, or SEN7-bR **b**, at concentrations of 1 nM for the stated times (●). The kinetics of protein synthesis inhibition by unconjugated PE or bR is shown for comparison (○). [$^3$H]leucine was present in the cultures for at least 1 h. Data are presented as the incorporation of [$^3$H]leucine as a percentage of untreated controls; lines bars, s.d.

**Figure 5** Cell-killing efficiency of SEN7 immunotoxins against SCLC cells in tissue culture. SW2 cells (○, ●) or LX1 control cells (□, ■) were incubated in the continuous presence of SEN7-PE (open symbols) or SEN7-bR (closed symbols) in limiting dilution clonogenic assays. Data are presented as surviving fractions, which were calculated by comparison with diluent-treated control cultures.

**Table III** Relative levels of SEN7 and SEN36 binding and cytotoxic activities of SEN7 immunotoxins and unconjugated toxins against clonogenic SW2 cells pre-exposed to the immunotoxins in limiting dilution assays

| *SW2 cells pre-exposed to*[a] | *Relative MFI SEN7/SEN36* | *$IC_{50}$*[b] *(M)* SEN7-PE | *SEN7-bR* | *PE* | *bR* |
|---|---|---|---|---|---|
| – | 325/378 | $(1.5 \pm 0.4) \times 10^{-11}$ | $(7.5 \pm 2.3) \times 10^{-12}$ | $(5.3 \pm 2.0) \times 10^{-10}$ | $(8.4 \pm 2.9) \times 10^{-10}$ |
| SEN7-PE | 333/371 | $(1.9 \pm 1.1) \times 10^{-9}$ | $(8.2 \pm 2.7) \times 10^{-12}$ | $(4.9 \pm 2.2) \times 10^{-9}$ | $(7.4 \pm 2.1) \times 10^{-10}$ |
| SEN7-bR | 98/109 | $(5.3 \pm 2.8) \times 10^{-9}$ | $(8.5 \pm 1.9) \times 10^{-10}$ | $(5.9 \pm 2.4) \times 10^{-10}$ | $(9.0 \pm 3.2) \times 10^{-10}$ |

[a]Cells pretreated with immunotoxins in limiting dilution assays. [b]Mean ± s.d. from three independent experiments in terms of toxin concentration. MFI, mean fluorescence intensity (average channel value).

escence intensities after indirect staining with SEN7 or SEN36, indicating that identical amounts of NCAM were expressed on their surfaces. Compared with the control cells, SEN7-PE pretreated cells were equally sensitive to SEN7-bR and unconjugated bR, but more than 100- and 10-fold less susceptible to SEN7-PE and PE intoxication respectively. In contrast, SW2 cells which had survived pretreatment with SEN7-bR in limiting dilution assays did not stain significantly with the antibodies, indicating that NCAM-deficient tumour cells or cells expressing very low levels of antigen (below the detection threshold of the assay) had been selected in the cultures. Accordingly, these cells were not selectively killed by either immunotoxin but were equally sensitive to unconjugated PE or bR compared with the control cells. Reexpression of NCAM gradually occurred during a time interval of 6 weeks in tissue culture in the absence of selective pressure (data not shown).

### Discussion

Ricin A-chain immunotoxins directed against antigens of the clusters w4 and 5a have shown substantial and selective cytotoxic and therapeutic potency against SCLC cells in preclinical studies *in vitro* and *in vivo* (Wawrzynczak *et al.*, 1990; 1991; Derbyshire *et al.*, 1992*a*; Zangemeister-Wittke *et al.*, 1993*a*,*b*). In contrast, ricin A-chain immunotoxins directed against the cluster 1 antigen which corresponds to the neural cell adhesion molecule (NCAM) could not prove potent cytotoxic agents (Wawrzynczak *et al.*, 1991; Derbyshire *et al.*, 1992*a*). This is consistent with our finding that NCAM, which was complexed on the target cell surface with the MAb SEN7, was not well internalised and remained on the cell surface. Immunotoxins act on intracellular targets, consequently internalisation is a major requirement for their cytotoxic activity. We have coupled the whole toxin molecules of native ricin or *Pseudomonas* exotoxin A (PE) to the MAb SEN7 because these toxins possess the ability to promote internalisation of the conjugates via their natural receptors on the cell surface. In order to warrant a high degree of selectivity the binding activities of the toxins were substantially reduced. With ricin this was achieved by covalently blocking the galactose binding sites of the B-chain with affinity ligands, resulting in a blocked ricin molecule (bR) with a more than 3,500-fold lower binding affinity and a 1,000-fold lower non-specific toxicity compared with native ricin (Lambert *et al.*, 1991*a*,*b*). Native PE was conjugated to SEN7 via a thioether linkage (Morgan *et al.*, 1990; FitzGerald *et al.*, 1990). Both SEN7-bR and SEN7-PE are immunologically distinct molecules inhibiting protein synthesis in cells by acting on different cellular targets. Therefore, their combination could help to (1) circumvent neutralising immune responses directed against the toxin moiety and (2) achieve higher cytotoxic potency against tumour cells with different sensitivities to different toxins. Whereas the susceptibility of SCLC cell lines to ricin immunotoxins is well documented, immunotoxins against SCLC which employ the toxic moiety of PE have not been previously described. SEN7-bR and SEN7-PE were potently and selectively active against various SCLC cell lines of both classic and variant morphologies with $IC_{50}$ values ranging between 7 and 85 pM. With each immunotoxin, intoxication of target cells proceeded rapidly following a 2 h lag phase. The potency of the bR conjugate was approximately 1 log higher than that of the PE conjugate as judged by the unspecific killing of antigen-negative tumour cells. The latter, on the other hand, was more selective. Although binding of the toxin moieties to their natural receptors efficiently promoted internalisation and cytotoxic action of the SEN7 immunotoxins, the unconjugated toxins alone were significantly less effective. One might speculate that binding of SEN7 to NCAM directs the toxins in close proximity to their natural receptors and induces the formation of high-affinity receptor–ligand complexes which are more likely to be rapidly and efficiently transported to the translocation compartment.

The cytotoxic effects of the ricin immunotoxins could be drastically enhanced by monensin. Potentiation of SEN7–ricin A-chain, in particular, indicates that even in the absence of the B-chain at least a small proportion of the NCAM epitope was internalised by the cells, albeit in a fashion not conducive to efficient A-chain translocation to the cytosol. The extreme cytotoxic activity and the lower rate of potentiation of SEN7-bR by monensin suggest that the B-chain alone is sufficient to fulfil the requirements for efficient intoxication of target cells. In contrast to the ricin immunotoxins, monensin inhibited the action of SEN7-PE. This might reflect the difference in the translocation compartments for the catalytic subunits of ricin and PE. Whereas ricin translocates in the trans-Golgi network (Olsnes *et al.*, 1989; Sandvig *et al.*, 1991), translocation of PE seems to be confined to the endoplasmic reticulum (FitzGerald & Pastan, 1992). In view of recent progress in enhancing the potentiating effect of monensin *in vivo* (Hertler *et al.*, 1989; Griffin *et al.*, 1993), ricin derivatives might prove advantageous agents in cases where immunotargeting is directed against non-internalising cell-surface antigens. The lack of potentiation of SEN7 immunotoxins by lysosomotropic amines suggests that lysosomal degradation was not a factor limiting the cytotoxic potency of these agents.

Despite the stable and frequent expression of NCAM on most SCLC cells, NCAM-deficient tumour cells or at least cells expressing very low levels of antigen emerged under the selection pressure exerted by SEN7-bR. Re-expression of NCAM occurred during a time interval of 6 weeks in tissue culture in the absence of the immunotoxin, indicating that antigen deficiency was acquired in response to the selection process. In contrast, cells pretreated with SEN7-PE fully retained their NCAM expression pattern and their sensitivity to SEN7-bR but were more than 10-fold less susceptible to intoxication by PE. The different mechanisms of resistance which were observed following pretreatment with the immunotoxins coincide with the different mechanisms of action of the corresponding catalytic domains. Ricin has an *N*-glycosidase activity which is not reversible (Endo *et al.*, 1987). Similarly, the ADP-ribosyl transferase activity of PE is essentially irreversible under physiological conditions (Iglewski *et al.*, 1977). However, resistance to PE can emerge as a result of the selection of elongation factor 2 variants which are less accessible to the toxin. Further mechanisms which might contribute to the resistance of SEN7-PE-pretreated cells include inherent differences in their ability to internalise and process the immunotoxin and cell-surface alterations that impede the passage of the toxin across the membrane (Godal *et al.*, 1986; Goldmacher *et al.*, 1987). The capability of SCLC cells to modulate prominent cell-surface antigens during antibody-based immunotherapy and to acquire resistance to toxins and chemotherapeutic drugs strongly supports their high evolutionary potential. As a rationale, a combination of alternative therapeutic strategies should include immunotoxins which employ not only different toxins but also different target antigens in order to achieve maximal efficacy. Altogether, since the SCLC cells were less able to develop resistance to ricin as compared with PE, ricin seems to be a more potent cytotoxic agent in this tumour system.

In conclusion, we describe the potent and selective cytotoxic activities of immunotoxins made with the MAb SEN7, recognising a new NCAM epitope on SCLC cells. The lack of significant spontaneous endocytosis of cell surface-bound SEN7 implies that in order to make a selective and potent immunotoxin against NCAM the toxin moiety must be capable of promoting internalisation and translocation by weakened receptor–ligand interactions on the cell surface. This requirement is fulfilled by bR and by PE when coupled to the MAb via a thioether linkage.

We would like to thank Dr M. Kressel for performing laser microscopy. This work was supported by the Robert Wenner Award of the Swiss Cancer League and the Krebsforschung Schweiz.

### References

BLAKEY, D.C. & THORPE, P.E (1988). An overview of therapy with immunotoxins containing ricin or its A-chain. *Anti. Immuno. Radiopharma.*, **1**, 1-16.

CARBONE, D.P., KOROS, A.M.C., LINNOILA, R.I., JEWETT, P. & GAZDAR, A.F. (1991). Neural cell adhesion molecule expression and messenger RNA splicing patterns in lung cancer cell lines are correlated with neuroendocrine phenotype and growth morphology. *Cancer Res.*, **51**, 6142-6149.

CARNEY, D.N. & DE LEI, L. (1988). Lung cancer biology. *Semin. Oncol.*, **3**, 199-214.

CHANG, K., PAI, L.H., BATRA, J.K., PASTAN, I. & WILLINGHAM, M.C. (1992). Characterization of the antigen (CAK1) recognized by monoclonal antibody K1 present on ovarian cancers and normal mesothelium. *Cancer Res.*, **52**, 181-186.

CUMBER, A.J., FORRESTER, J.A., FOXWELL, B.M.J., ROSS, W.C.J. & THORPE, P.E. (1985). Preparation of antibody-toxin conjugates. *Methods Enzymol.*, **112**, 207-225.

DERBYSHIRE, A.J., STAHEL, R.A. & WAWRZYNCZAK, E.J. (1992*a*). Cytotoxic properties of a ricin A-chain immunotoxin recognising the cluster-5A antigen associated with human small-cell lung cancer. *Cancer Immunol. Immunother.*, **35**, 417-420.

DERBYSHIRE, E.J., STAHEL, R.A. & WAWRZYNCZAK, E.J. (1992*b*). Potentiation of a weakly active ricin A-chain immunotoxin recognizing the neural cell adhesion molecule. *Clin. Exp. Immunol.*, **89**, 336-340.

ENDO, Y., MITSUI, K., MOTIZUKI, M. & TSURUGI, K. (1987). The mechanism of action of ricin and related toxic lectins on eukaryotic ribosomes. *J. Biol. Chem.*, **262**, 5908-5913.

FITZGERALD, D., WILLINGHAM, M., CARDARELLI, C.O., HAMADA, H., TSURUO, T., GOTTESMAN, M.M. & PASTAN, I. (1987). A monoclonal antibody-pseudomonas toxin conjugate that specifically kills multidrug-resistant cells. *Proc. Natl Acad. Sci. USA*, **84**, 4288-4292.

FITZGERALD, D., IDZIOREK, T., BATRA, J.K., WILLINGHAM, M. & PASTAN, I. (1990). Antitumour activity of a thioether-linked immunotoxin: OVB3-PE. *Bioconjugate Chem.*, **1**, 264-268.

FITZGERALD, D.J. & PASTAN, I. (1992). Pseudomonas exotoxin: recombinant conjugates as therapeutic agents. *Biochem. Soc. Trans.*, **20**, 731-733.

GODAL, A., FODSTAD, O., MORGAN, A.C. & PIHL, A. (1986). Human melanoma cell lines showing striking inherent differences in sensitivity to immunotoxins containing holotoxins. *J. Natl Cancer Inst.*, **77**, 1247-1253.

GOLDMACHER, V.S., ANDERSON, J., SCHULZ, M.L., BLÄTTLER, W.A. & LAMBERT, J.M. (1987). Somatic cell mutants resistant to ricin, diphtheria toxin, and to immunotoxins. *J. Biol. Chem.*, **262**, 3205-3209.

GRIFFIN, T., RYBAK, M.E., RECHT, L., SINGH, M., SALINI, A. & RASO, V. (1993). Potentiation of antitumour immunotoxins by liposomal monensin. *J. Natl Cancer Inst.*, **85**, 292-298.

HERTLER, A.A., SCHLOSSMAN, D.M., BOROWITZ, H.J., BLYTHMAN, H.E., CASELLAS, P. & FRANKEL, A.E. (1989). An anti-CD5 immunotoxin for chronic lymphocytic leukemia: enhancement of cytotoxicity with human serum albumin-monensin. *Int. J. Cancer*, **43**, 215-219.

IGLEWSKI, B.H., LIU, P.V. & KABAT, D. (1977). Mechanism of action of pseudomonas aeruginosa exotoxin A: adenosine diphosphate-ribosylation of mammalian elongation factor 2 in vitro and in vivo. *Infect. Immun.*, **15**, 138-144.

JOHNSON, E.A. & BROWN, B.W. (1961). The spearman estimator for serial dilution assays. *Biometrics*, **30**, 79-88.

KOUNNAS, M.Z., MORRIS, R.E., THOMPSON, M.R., FITZGERALD, D.J., STRICKLAND, D.K. & SAELINGER, C.B. (1992). The $\alpha_2$-macroglobulin receptor/low density lipoprotein receptor-related protein binds and internalizes pseudomonas exotoxin A. *J. Biol. Chem.*, **267**, 12420-12423.

LAMBERT, J.M., MCINTYRE, G., GAUTHIER, M.N., ZULLO, D., RAO, V., STEEVES, R.M., GOLDMACHER, V.S. & BLÄTTLER, W.A. (1991*a*). The galactose binding sites of the cytotoxic lectin ricin can be chemically blocked in high yield with reactive ligands prepared by chemical modification of glycopeptides containing triantennary N-linked oligosaccharides. *Biochemistry*, **30**, 3234-3247.

LAMBERT, J.M., GOLDMACHER, V.S., COLLINSON, A.R., NADLER, L.M. & BLÄTTLER, W.A. (1991*b*). An immunotoxin prepared with blocked ricin: a natural plant toxin adapted for therapeutic use. *Cancer Res.*, **51**, 6236-6242.

LYNCH, T.J., CORAL, F., SHEFNER, J., ELIAS, A.D., SKARIN, A., MENTZER, S., SUGARBAKER, D., EPSTEIN, C., BLÄTTLER, W.A., COLLINSON, A.R. & RITZ, J. (1993). Phase one trial of the novel immunotoxin N901-blocked ricin (N901-BR): demonstration of clinical activity in small cell lung cancer. *ASCO*, **12**, 293.

MOOLENAAR, C.E., MULLER, E.J., SCHOL, D.J., FIGDOR, C.G., BOCK, E., BITTER, S.D. & MICHALIDES, R.J. (1990). Expression of neural cell adhesion molecule-related sialoglycoprotein in small cell lung cancer and neuroblastoma cell lines H69 and CHP-212. *Cancer Res.*, **50**, 1102-1106.

MORGAN, A.C., SIVAM, G., BEAUMIER, P., MCINTYRE, R., BJORN, M. & ABRAMS, P.G. (1990). Immunotoxins of pseudomonas exotoxin A (PE): effect of linkage on conjugate yield, potency, selectivity and toxicity. *Mol. Immunol.*, **27**, 273-282.

OLSNES, S., SANDVIG, K., PETERSEN, O.W. & VAN DEURS, B. (1989). Immunotoxins - entry into cells and mechanisms of action. *Immunol. Today*, **10**, 291-295.

PASTAN, I. & FITZGERALD, D. (1991). Recombinant toxins for cancer treatment. *Science*, **254**, 1173-1177.

PATEL, K., BOURNE, S., PHIMISTER, B., COAKHAM, H. & KEMSHEAD, J.T. (1990). Presence of neural cell adhesion molecule on human embryonic and brain tumours. *Biochem. Soc. Trans.*, **18**, 408-410.

ROTHBARD, J.B., BRACKENBURY, R., CUNNINGHAM, B. & EDELMAN, G. (1982). Differences in the carbohydrate structures of neural cell adhesion molecules from adult and embryonic chicken brains. *J. Biol. Chem.*, **257**, 11064-11069.

ROUGON, G., DEAGOSTINI-BAZIN, H., HIRN, M. & GORIDIS, C. (1982). Tissue and development stage specific forms of neural cell surface antigen linked to differences in glycosylation of a common polypeptide. *EMBO J.*, **1**, 1239-1244.

SANDVIG, K., PRYDZ, K., HANSEN, S.H. & VAN DEURS, B. (1991). Ricin transport in brefeldin A-treated cells: correlation between golgi structure and toxic effect. *J. Cell Biol.*, **115**, 971-981.

SHAH, S.A., HALLORAN, P.M., FERRIS, C.A., LEVINE, B.A., BOURRET, L.A., GOLDMACHER, V.S. & BLÄTTLER, W.A. (1993). Anti-B4-blocked ricin immunotoxin shows therapeutic efficacy in four different SCID mouse tumor models. *Cancer Res.*, **53**, 1360-1367.

SOUHAMI, R.L., BEVERLEY, P.C.L., BOBROW, L.G. & LEDERMANN, J.A. (1991). Antigens of lung cancer: results of the second international workshop on lung cancer antigens. *J. Natl Cancer Inst.*, **83**, 609-612.

WAIBEL, R., MANNHART, M., O'HARA, C.J., BROCKLEHURST, C., ZANGEMEISTER-WITTKE, U., SCHENKER, T., LEHMANN, H.P., WEBER, E. & STAHEL, R.A. (1993). Monoclonal antibody SEN7 recognizes a new epitope on the neural cell adhesion molecule on small cell lung cancer but not on lymphocytes. *Cancer Res.* (in press).

WAWRZYNCZAK, E.J., DERBYSHIRE, E.J., HENRY, R.V., PARNELL, G.D., SMITH, A., WAIBEL, R. & STAHEL, R.A. (1990). Selective cytotoxic effects of a ricin A-chain immunotoxin made with the monoclonal antibody SWA11 recognising a human small cell lung cancer antigen. *Br. J. Cancer*, **62**, 410-414.

WAWRZYNCZAK, E.J., DERBYSHIRE, E.J., HENRY, R.V., PARNELL, G.D., SMITH, A., WAIBEL, R. & STAHEL, R.A. (1991). Cytotoxic activity of ricin A-chain immunotoxins recognising cluster 1, w4 and 5A antigens associated with human small cell lung cancer. *Br. J. Cancer*, **63**, 71-73.

ZANGEMEISTER-WITTKE, U., LEHMANN, H.P., WAIBEL, R., WAWRZYNCZAK, E.J. & STAHEL, R.A. (1993*a*). Action of a CD24-specific deglycosylated ricin-A-chain immunotoxin in conventional and novel models of small-cell-lung-cancer xenograft. *Int. J. Cancer*, **53**, 521-528.

ZANGEMEISTER-WITTKE, U., COLLINSON, A.R., FISCH, I., JONES, R.M.L., WAIBEL, R., LEHMAN, H.P. & STAHEL, R.A. (1992*b*). Anti-tumor activity of a blocked ricin immunotoxin with specificity against the cluster-5A antigen associated with small-cell lung cancer. *Int. J. Cancer*, **54**, 1028-1035.